# Alcohol as a Remedy for the Bite of the Rattlesnake

**Published:** 1853-01

**Authors:** W. K. Hopkins

**Affiliations:** Fox Lake, Wis.


ARTICLE III.—*Alcohol as a Remedy for the Poison of the Rattlesnake*, by DR. W. K. HOPKINS, FOX LAKE, WIS.

IN the Nov. number of the North-Western Medical and Surgical Journal, I read an article on the use of Alcohol as a remedy for the poison of the Rattlesnake. On the mountain-tops of Northern Pennsylvania and Southern New York, the Rattlesnake, the most venomous of its kind in our northern latitudes, is frequently met with. Deaths were not unfrequent; but the accidental discovery that Brandy was a sure antidote, has lessened the fears of the inhabitants of at least a portion of that section, for they find, when timely administered, that it cures in every instance. I speak confidently from my own experience.

I will give you two cases; one occurring in Bradford County, Pennsylvania, the other in the town of Fox Lake, Dodge County, Wisconsin.

In 1840, near mid-summer, I was called to visit W. P., reported to have been bitten by a large Rattlesnake, in the town of Ridgebury, Pa. On my arrival, I learned from his friends that he was bitten near a mile from home, on the hill-side, and that his companions corded the limb and hurried him to his house. He was a blacksmith by trade, quite dissipated, and now very much alarmed. The leg was very much swollen below the ligature. He complained of great pain in and about the wound, which was an inch or more above the inner ankle, a mere puncture, as if done with a needle. His pulse feeble and intermitting; dimness of vision; nausea, with slight vomiting; the color of the leg growing more livid.

I found he had drank no spirits that day (Sunday,) and I ascribed, in part, his prostration to that cause, knowing his habits, and to his extreme fright. I consequently ordered him half a wine glass of Brandy; in five minutes I repeated it, carefully observing its effects. In ten minutes I gave him a full wine glass, and in twenty minutes, finding his pulse much stronger, more regular, and the countenance losing much of its horror, I again repeated the dose of Brandy, a full glass. In the meantime, however, I had removed the ligature from the limb, and cauterized the wound with Nit. Argent., having previously made an incision to the bottom of the wound with a lancet.

It is important, when we depend on external applications, to reach the bottom of the puncture made by the tooth, as it is a well known fact that the poisoned fang is tubular, and the virus is injected through it into the extreme depth reached by the tooth.

It was near two o'clock P. M. when I reached my patient, and by six o'clock he had taken a full pint of Brandy. I gave directions that the limb be washed in cold vinegar and water, and to give him Brandy every two hours, until I should see him. He now expressed confidence in his recovery; he said his vision was clear, his faintness had left him, and the nervous energy, at first much prostrated, was in a measure restored. The leg, however, was much tumefied, but not painful; the swelling had not extended.

In about eighteen hours I visited my patient again, and found he had passed the night sleeping more than half the time; somewhat thirsty, slight nausea, but he was sitting up, and said he felt quite well. The swelling of the limb had very much subsided; his vision remained perfect; pulse regular, although accelerated. I gave him a tonic, added the Acetate of Lead to the lotion for the limb, and left him. The next Saturday he visited our village, as well as ever.

I have had one case in this neighborhood. The daughter of A. H., a young girl of ten or eleven years old, was bitten by a Rattlesnake, just forward of the inner ankle. The parents had corded the limb, and had to send five miles for aid. Taking sometime to obtain a messenger, it was at least two hours after the accident before I saw my little patient. Not to be too prolix; all the symptoms of the virus taking effect were present—swollen limb, feeble fluttering pulse, great nervous prostration, dimness of vision, &c. It took another half hour before we obtained Brandy. I finally gave her a large table-spoonful every ten minutes, until she was fully under its influence, and it was remarkable to see, after taking three or four doses, the change in the action of the heart. No doubt dependent on the energy given to the nervous system, the pulse became more regular and fuller; the countenance, before flushed and anxious, became calm in its expression; the child, when I arrived, could not distinguish one from another across the room; the vision was improved, although yet imperfect. I repeated the Brandy from time to time, at intervals of thirty or forty minutes. I made the usual external applications, cauterized the wound, &c. Next day I found her out of danger. She could walk from the bed to a chair without pain or difficulty, although the limb was slightly tumefied. In a few days she had perfectly recovered.

I think all the symptoms attending the bite of the Rattlesnake can be accounted for from its prostrating effects on the nervous system; maintain the sensorial power, and you overcome the consequences of the venom.

